# Measuring social participation in children with chronic health conditions: validation and reference values of the child and adolescent scale of participation (CASP) in the German context

**DOI:** 10.1186/s12887-019-1495-6

**Published:** 2019-04-24

**Authors:** Freia De Bock, Catherin Bosle, Christine Graef, Johannes Oepen, Heike Philippi, Michael S. Urschitz

**Affiliations:** 1 Division of Paediatric Epidemiology, Institute of Medical Biostatistics, Epidemiology und Informatics, University Medical Centre of the Johannes Gutenberg-University, Langenbeckstraße 1, 55101 Mainz, Germany; 2 Mannheim Institute of Public Health, Social and Preventive Medicine, University Medicine Mannheim, Heidelberg University, Ludolf-Krehl Strasse 7-11, 68167 Mannheim, Germany; 3 Klinik Viktoriastift, Cecilienhöhe 3, 55543 Bad Kreuznach, Germany; 4 Center for Child Neurology, Frankfurt-Mitte, Theobald-Christ-Str. 16, 60316 Frankfurt am Main, Germany

**Keywords:** Social participation, Children, Chronic health condition, Quality of life, Validation, Psychometric evaluation

## Abstract

**Background:**

While ICF-CY-based models of care are promising avenues for improving participation of children with chronic health conditions, feasible and valid instruments to assess participation as an outcome in routine are still needed. We aimed to validate a German parent-report version of the Child and Adolescent Scale of Participation (CASP) in children with chronic health conditions of different severity.

**Methods:**

Cross-sectional data were collected in 327 children (mean age 7.8 years, 55% boys) from two paediatric centres (*n* = 112) and one population-based sample (*n* = 215). Cronbach’s alpha, factor analyses, face validity assessments, correlation analyses, receiver operating characteristics (ROC) curves, and parent-reported health-related quality of life (HRQoL: KINDL) were used to examine internal consistency, test-retest reliability, and capacity to differentiate between disease severity groups. Disease severity was operationalized according to ICD-diagnosis groups and/or parent-reports on health problems, medical and educational support, and medication. A newly developed item “overall perceived participation” was added to the CASP and evaluated.

**Results:**

We found good to excellent content validity, excellent internal consistency, and good-to-excellent test-retest reliability of the instrument. While children with mild disease had a significantly greater extent of participation (higher CASP scores) than children with severe disease, they did not differ from healthy children. Children with mild compared to severe disease much more differed in participation as measured by the CASP compared to the KINDL (area under the ROC curve: 0.92 vs. 0.75). In addition, the item “overall perceived participation” was highly correlated (r = 0.86) with the CASP total score, indicating the potential value of this specific single item. Finally, we provided preliminary reference values for the CASP obtained in a population-based sample of children without chronic health conditions.

**Conclusions:**

The German version of the CASP and the new item are efficient, valid and reliable measures of social participation in childhood. The CASP-measured participation focuses more on attendance than on involvement into social circumstances of everyday life. To detect children with a high burden of disease on everyday life, the CASP may be more accurate than HRQoL instruments such as the KINDL. As outcome measurement, the CASP may facilitate the implementation of patient-centred paediatric health care.

**Electronic supplementary material:**

The online version of this article (10.1186/s12887-019-1495-6) contains supplementary material, which is available to authorized users.

## Background

The prevalence of chronic health conditions in children has dramatically increased in the last decades [1]. Besides a rise of socially determined “new morbidities” (e.g. attention-deficit/hyperactivity-disorder, obesity), improved survival of perinatal, genetic and metabolic conditions (e.g. preterm birth, cerebral palsy, cystic fibrosis) explains this trend [2]. However, only few of these conditions resolve spontaneously or can be cured completely. Thus, the development of new, long-term, patient-oriented models of care will be a key challenge for the future [3]. In addition, outcomes other than having a diagnosis or not (e.g. ICD-10 Code [4]) or being free of symptoms are needed to guide future paediatric health care services.

The World Health Organization anticipated this development by publishing the International Classification of Functioning, Disability and Health-Children and Youth (ICF-CY) in 2007 [5]. This classification scheme aims at establishing a universal language to improve quality and relevance of health care services for children with chronic health conditions and/or disability. Besides physical and mental functioning, the ICF-CY classifies contextual factors, activities, and - as an ultimate goal of medical care – (social) participation. Although the ICF-CY was published more than 10 years ago, systematic reviews showed that application and practical implementation of the ICF-CY is very limited in paediatric health care services and deserves more efforts [6].

Within the ICF-CY, participation is broadly defined as “problems an individual may experience in involvement in life situations” and its theoretical construct covers 9 distinct domains [5, 7]. However, the distinction between impaired functioning, activities, participation, and quality of life is blurred and actual instruments do not clearly distinguish between these constructs according to recent WHO definitions [8]. In particular, the concept of participation and its constructs according to the ICF-CY are only insufficiently transferred into scientific practice. In a recent systematic review evaluating methods in participation intervention research, five different themes of participation could be identified in 25 articles analysed [9]. Two themes, attendance and involvement, were directly related to participation, while three themes were only related concepts (i.e. preferences, activity competence, and sense of self; [9]. This led to the proposition of a “family” of participation-related constructs, where attendance and involvement seem to describe the essence of the participation concept while the related themes such as preferences and activity competence are rather important means to enhance participation [9].

With the ICF-CY defining participation as an ultimate goal and outcome measure of health care services, feasible, acceptable, valid, and cost-effective instruments to assess activities and participation in routine care are needed. A recent review on children’s participation measures showed that most instruments came from the English-language literature, were rather long (mean number of items: 37), and assessed participation only with some items (mean proportion of items covering participation: 49%; [10]). Despite the ICF-CY being the conceptual basis for these instruments, only the Child and Adolescent Scale of Participation (CASP; 20 items; [11]) and the Participation and Environment Measure for Children and Youth (PEM-CY; 58 items; [12]) cover all nine domains of activities and participation as specified in the ICF-CY. Of the 20 CASP items, 1, 6, 4, 7, 9, 9, 2, 11, and 20 items cover the ICF-CY domains d1, d2, d3, d4, d5, d6, d7, d8, and d9, respectively [10]. 25 and 75% of the items cover activities and participation, respectively. Activities and participation are assessed in the areas “home”, “community”, “school”, and “living activities”. The CASP may be seen as participation-centred outcome instrument and may be valuable for participation intervention research.

Thus, we aimed to establish a culturally adapted German version of the CASP to promote participation as an outcome measure of daily health care and health care services research in the German language area. Therefore, we applied it to parents of children with and without a wide range of chronic health conditions of different severity and assessed its validity and reliability. As pointed out by Rainey et al. [13], existing instruments (e.g. from the Anglo-American area) should be culturally adapted and validated before its use in contexts that differ relevantly in terms of culture, health care and/or educational system.

## Methods

### Settings and participants

This study took place in three different settings resulting in two clinical and one population-based samples: i) an outpatient paediatric neurology centre (clinical sample 1; SPZ Frankfurt/Main, Federal State of Hesse, Germany), ii) an inpatient paediatric rehabilitation clinic (clinical sample 2; Klinik Viktoriastift Bad Kreuznach, Federal State of Rhineland-Palatinate, Germany) and iii) within the city limits of Mainz and the rural district of Mainz-Bingen (population-based sample; Federal State of Rhineland-Palatinate, Germany). This approach was chosen to i) cover a broad range of chronic conditions, living environments, and health care services and ii) test the CASP’s ability to differentiate between children with and without chronic health conditions of various severities.

The clinical sample 1 consisted of children with predominantly chronic neuro-paediatric conditions (e.g. cerebral palsy, epilepsy, neuro-genetic conditions) and was recruited via convenience sampling at regularly scheduled appointments at the outpatient centre between November 2015 and April 2016. The clinical sample 2 was a convenience sample of children and adolescents with a broad spectrum of different chronic health conditions such as diabetes, obesity, and attention-deficit/hyperactivity-disorder (ADHD), who were recruited at the inpatient clinic between March and June 2016. The population-based sample consisted of participants of an ongoing prospective cohort study of children officially registered for school entry in autumn 2014 [14]. Recruitment for the current study was performed at the end of first grade in June 2015.

Inclusion criteria for the clinical samples were: 3–11 years of age, a certified medical diagnosis, and sufficient German language skills of parents and children. Inclusion criterion for the population-based sample was active participation at the time of recruitment in the underlying cohort study. Written informed consent was obtained in parents and in children of more than 8 years of age. The study was approved by the Medical Ethics Review Board of the Medical Faculty Mannheim at Heidelberg University (2015-550 N-MA), the Ethics Committee of the Federal Physician Chamber of Hesse (MC180/2015) and the Ethics Committee of the Regional Medical Association of Rhineland-Palatinate (837.544.13 [9229]).

### Study design and procedures

A prospective validation study was performed. After instrument translation, validity and reliability was assessed in using cross-sectional (validity) and longitudinal (reliability) study designs. Data were largely obtained by questionnaires, which were either handed out in person (e.g. at regular appointments in the clinical samples) or mailed to parents in the population-based sample. Parents took about 10–15 min to fill in the questionnaires and were asked to hand back the questionnaires after the appointment or send it back to the study centre using prepared stamped envelopes. Beyond this survey, detailed information on chronic health conditions was retrieved either from the local clinical information system (clinical samples) or from the preschool examination database (population-based sample) provided by the regional Department of Public Health (County Government Mainz-Bingen).

### Assessment of social participation

Social participation was measured using a German version of the CASP [11]. The CASP is a 20-items 4-scales parent-report instrument covering attendance and involvement in the areas “home”, “community”, “school”, and “living activities”. The instrument measures the extent to which children of 3 to 18 years of age participate in activities in comparison to healthy children of the same age. Due to its shortness, however, the CASP does not differentiate between different aspects of participation such as attendance and involvement. Responses are rated on a 4-point scale ranging from 4 “age expected” “(i.e. full participation)” and 3 “somewhat limited” to 2 “very limited” and 1 “unable to participate”. An additional fifth response category of “not applicable” is provided for all items. In addition, the instrument offers three open-ended questions that can be used for individualized family-centred care planning. The original CASP was shown to have high reliability (Intra-class coefficient = 0.94; Cronbach’s α > 0.96) and usefulness to discriminate between children with and without disabilities [11, 15]. Scores are summed up and calculated for the total scale and 4 subscales [16]. The scores of the scales range from 0 to 100%, higher scores thereby indicate more favourable participation.

The original English version was translated and back-translated by two independent translators as requested by international standards [17]. Based on comparisons of translated versions, a final version was consented after discussion among translators and an additional expert in the field (HP). This version was then applied to seven parents and nine experts in the field to assess cultural and content appropriateness. Based on the feedback, further cultural and language adaptations were undertaken to increase acceptance in German language settings. Cultural adaptions encompassed primarily a change of illustrative examples for activities as well as the use of simplified vocabulary to make the items easier to understand for parents with migrant background. For example, leisure-time physical (German: “Kinderturnen”) and musical education (German: “Musikschule”) were added as important additional after-school activities.

### Assessment of chronic health conditions

The identification of chronically ill children was based on the following data available: i) ICD-10 codes from the clinical information systems (clinical samples), ii) parent-reported doctor diagnoses from the preschool examination database (population-based sample), and iii) information on special health care needs as assessed in the preschool examination of Rhineland-Palatine (all three samples).

As there was no international definition for the identification and severity rating of chronic health conditions, we developed an algorithm based on the criteria published by Perrin et al. [18] and the ICF principals. Ratings were performed independently by two medical experts (FDB (author, pediatrician), CS (speech therapist)) and reviewed by a third one (HP (author, pediatrician, pediatric neurologist)). The following chronic health conditions were considered: combined developmental problems, obesity, ADHD, behavioral and conduct problems, cerebral palsy, epilepsy, autism, genetic syndromes, intellectual disability, and former cancer.

**Fig. 1:**
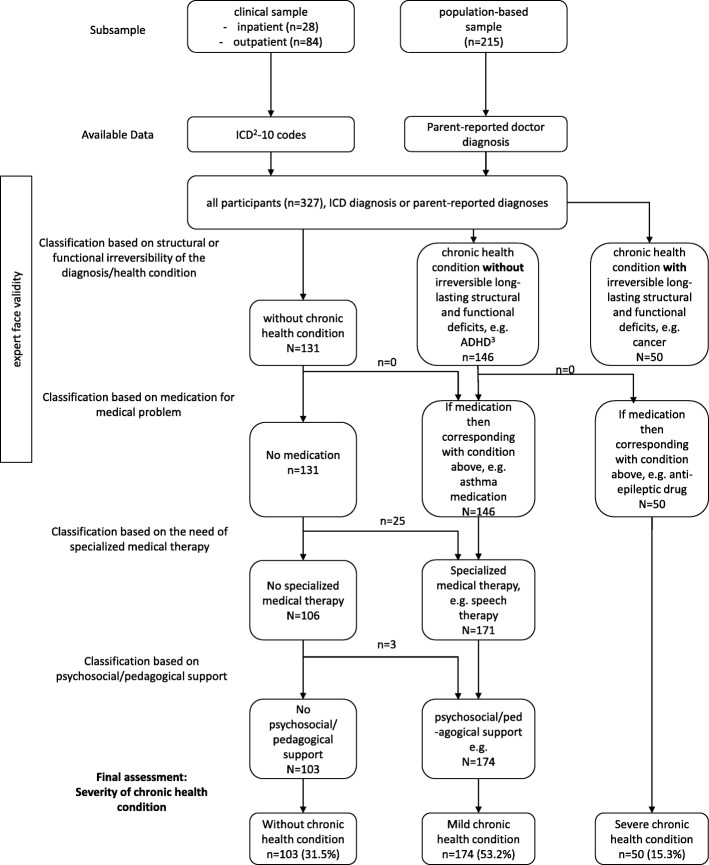
Flow chart on the operationalization of children with chronic health conditions and different disease severity (for detailed description of operationalization see methods)

First, presence of one of the above chronic health conditions was determined by reviewing the ICD-10 codes and parent-reported doctor diagnoses. Second, based on ICD-10 codes and doctor diagnoses, the health condition was rated as severe, if long-lasting, irreversible structural and functional deficits were present or could be expected. Otherwise, the condition was rated as mild. Third, needs and uptake of prescribed medication for a medical problem were evaluated. Depending on the presence of such a medication, the medical condition could be upgraded (e.g. from no to mild or mild to severe). In a fourth step, needs and uptake of physical, occupational, or speech therapy were evaluated. Depending on the presence of such a treatment, children were upgraded from “without chronic condition” to mild health condition, to also include conditions not yet recognized as diagnosis. Last, needs and uptake of social or educational support or mental health services were evaluated. Again, depending on the support, the children were upgraded from “without chronic condition” to mild health condition. The identification and rating steps of this algorithm are illustrated in Fig. 1. This algorithm allowed the combination of information of the condition itself with information the condition’s consequences and impact and the actual health care use.

### Assessment of health-related quality of life

Health-related quality of life (HrQoL) was measured using the KINDL^R^ parent-proxy instrument [19]. The KINDL^R^ is a 24-items 6-scales parent-reported instrument covering the domains “physical wellbeing”, “emotional wellbeing”, “self-esteem”, “family”, “peers”, and “pre-school/school”. Responses are rated on a five-point scale ranging from “never” and “rarely” to “sometimes”, “frequently” and “always”. Comparable to the CASP, a total scale (scores range from 0 to 100) and subscales are available. Results for validity and reliability of the instrument (Chronbach’s α = 0.85 for the total scale) have been published elsewhere [20].

Although the KINDL^R^ was developed as a measure of HrQoL, only 36% of its items were found to be congruent with the WHO HrQoL definition [8]. In contrast, more than 50% of the items were congruent with one of the ICF-CY domains [8]. We, hence, expected some positive relationships between parent-reported CASP and KINDL measures, although the concepts of HrQoL and participation are theoretically quite different.

### Assessment of socio-demographic factors

Age, sex, and migrant background of the child as well as parental education was obtained by additional questionnaire items. Parental education was defined based upon the highest parental level of education achieved, according to international standard classification of education (ISCED; high = ISCED level 4–5, medium = ISCED level 3, and low = ISCED levels 0–2). Migrant background was operationalized based on the recommendation by the German National Statistics Bureau [21], which accounts for parental nationality and country of birth.

### Validation process and statistical analysis

Descriptive statistics (n and percentage, mean and standard deviation) were used to describe the distribution of socio-demographic and clinical factors among samples and disease severity groups (Tables 1 and 2).

**Table 1:** Description of study population by sample

		Clinical sample 1		Clinical sample 2		Population-based sample		Total sample	
*N* (%)		84 (25.69)		28 (8.56)		215 (65.75)		327 (100.00)	
		*n* (%) or mean (SD)	*n* (%) missing values	*n* (%) or mean (SD)	*n* (%) missing values	*n* (%) or mean (SD)	*n* (%) missing values	*n* (%) or mean (SD)	*n* (%) missing values
Age (years)		6.21^***^ (1.94)	–	8.31^***^ (2.38)	–	7.27^***^ (0.32)	–	7.08 (1.36)	–
Sex	female	24^**^ (28.57)	–	10^**^ (35.71)	–	110^**^ (51.16)	–	144 (44.04)	–
Migrant background	yes	40^***^ (47.62)	18 (21.43)	3^***^ (10.71)	8 (28.57)	16^***^ (7.44)	8 (3.72)	59 (18.04)	34 (10.40)
Parental education	high	39^***^ (46.43)	5 (5.95)	4^***^ (14.29)	1 (3.57)	137^***^ (63.72)	4 (1.86)	180 (55.05)	10 (3.06)
	medium	36^***^ (42.86)		22^***^ (78.57)		68^***^ (31.63)		126 (38.53)	
	low	4^***^ (4.76)		1^***^ (3.57)		6^***^ (2.79)		11 (3.36)	
CASP		75.58*** (19.56)	–	91.70*** (9.32)	–	98.16*** (5.80)	–	91.80 (14.87)	–
HrQoL		73.77*** (9.81)	10 (11.90)	71.31*** (13.48)	–	83.30*** (8.02)	3 (1.40)	79.99 (10.24)	13 (3.98)

*CASP* Child and Adolescent Scale of Participation, *HrQoL* Health related Quality of Life, *SD* Standard deviation; *Age-adjusted values; † *p* < .05;

** *p* < .01; *** *p* < .001

**Table 2:** Description of study population by disease severity

		Without chronic health condition (healthy)		Mild chronic health condition		Severe chronic health condition		Total	
N (%)		103 (31.50)		174 (53.21)		50 (15.29)		327 (100.00)	
		*n* (%) or mean (SD)	*n* (%) missing values	*n* (%) or mean (SD)	*n* (%) missing values	*n* (%) or mean (SD)	*n* (%) missing values	*n* (%) or mean (SD)	*n* (%) missing values
Age (years)		7.23^†^ (0.29)	–	7.13^†^ (1.37)	–	6.61^†^ (2.18)	–	7.08 (1.36)	–
Sex	female	54^†^ (52.43)	–	75^†^ (43.10)	–	15^†^ (30.00)	–	144 (44.04)	–
Migrant background	yes	9^***^ (8.74)	3 (2.91)	31^***^ (17.82)	18 (10.34)	19^***^ (38.00)	13 (26.00)	59 (18.04)	34 (10.40)
Parental education	high	66 (64.08)	4 (3.88)	86 (49.43)	5 (2.87)	28 (56.00)	1 (2.00)	180 (55.05)	10 (3.06)
	medium	29 (28.16)		7 (44.25)		20 (40.00)		126 (38.53)	
	low	4 (3.88)		6 (3.45)		1 (2.00)		11 (3.36)	
CASP		99.11^***^ (2.48)	–	94.65 *** (8.77)	–	66.86 *** (20.26)	–	91.80 (14.87)	–
HrQoL		84.41*** (7.01)	1 (0.97)	78.83*** (10.83)	5 (2.87)	74.02*** (10.21)	7 (14.00)	79.99 (10.24)	13 (3.98)

*CASP* Child and Adolescent Scale of Participation, *HrQoL* Health related Quality of Life, *SD* Standard deviation, *Age-adjusted values, † *p* < .05;

** *p* < .01; *** *p* < .001

Content validity was assessed using a face-validity approach. Eight experts (2 paediatricians, 2 speech therapists, 2 physiotherapists, and 2 occupational therapists) rated comprehensibility, importance, and conceptual comprehensiveness of the instrument on a 5-point rating scale ranging from “not at all” to “very much”.

Exploratory factor analysis (EFA) using a promax rotation was performed to analyse the underlying structure of the CASP items. Due to missing values (mostly because parents chose the “not applicable” option), an imputation technique with an expectation-maximization (EM) algorithm was applied [22]. This method was found to provide a better basis for EFA than other more traditional methods (e.g. complete case analysis or mean imputation [23]). To specify nominal sample size, we use the column-wise minimum, which is defined as the number of complete cases for the variables with the most missing values [22]. The Kaiser criterion (eigenvalues > 1) was applied to define the number of factors. Finally, internal consistency of the CASP was analysed using Cronbach’s alpha for both, the total scale and all subscales.

In absence of a German reference standard for measuring participation, relationships between CASP and KINDL^R^ measures were investigated. Therefore, a single questionnaire item covering parental perceived overall social participation ranging from 1 “unable to participate” to 4 “age expected” was introduced and Pearson’s correlation coefficients between CASP scales, KINDL^R^ scales, and perceived overall participation were calculated.

Test-retest reliability was assessed by repeated administrations of the instrument in children from the clinical sample 1 with a time lapse of 2 to 4 weeks between measurements. Therefore, intra-class correlation (ICC) coefficients were calculated for the CASP total scale and all subscales.

CASP reference values were calculated based on the population-based sample and contrasted to the results of the clinical samples. The association between disease severity and participation was analysed in using linear regression analysis with the CASP total scale as the depending variable and disease severity, age, sex, and parental education as independent variables. The ability of the CASP to classify children according to their disease severity was investigated in using receiver operating characteristic (ROC) curves and area under this curve (AUC). Results for the CASP were contrasted to results for the KINDL. All statistical analyses were performed using STATA 13.1 [24].

## Results

### Demographic and clinical characteristics of the sample

In total, 327 children were enrolled into the present study (clinical sample 1: 84 children, clinical sample 2: 28 children, population-based sample: 215 children). Demographic characteristics stratified by sample are given in Table 1. In total, 103 children (31.5%) were classified as without chronic health condition, while 174 children (53.2%) were categorized as mild chronic health condition and 50 children (15.3%) as having a severe chronic health condition. Demographic characteristics stratified by disease severity are given in Table 2.

Here: Tables 1 and 2.

### Content validity

Comprehensibility of the final German CASP items (see Additional file 1) was rated to be 3.8 (i.e. 76% of a maximum of 5) for subscale “home”, 4 (80%) for subscale “community”, 4.7 (93%) for subscale “school”, and 4.6 (91%) for subscale “living activities”. Importance ratings were as follows: 4.7 (i.e. 93% of a maximum of 5), 4.6 (91%), 4.7 (93%) and 4.4 (89%) for all subscales, respectively. Finally, conceptual comprehensiveness was rated 4.4 (88% of a maximum of 5), 4.1 (82%), 4.2 (84%), and 4.6 (91%) for all the subscales, respectively. These results corresponded to good to excellent content validity.

### Underlying scale structure and internal consistency

Results for the EFA are provided in Table 3. The column-wise minimum was *n* = 290. The Kaiser criterion implied an underlying one-factor structure. This factor contributed to 88.7% of the variance explained. Cronbach’s alpha for both, the total scale (α = 0.98) as well as for the subscales A-D (A: α = 0.94, B: α = 0.92, C: α = 0.93, and D: α = 0.93) indicated excellent internal consistency.

**Table 3:** Exploratory factor analysis using a promax rotation (*n* = 290)

CASP items	Factor 1
Home: Social/leisure (family)	0.86
Home: Social/leisure (friends)	0.90
Home: chores/responsibilities	0.86
Home: Self-care	0.87
Home: Mobility	0.81
Home: Communication	0.85
Community: Social/leisure (friends)	0.86
Community: Structured activities	0.88
Community: Mobility	0.86
Community: Communication	0.88
School: Educational activities	0.80
School: Social/leisure (students)	0.86
School: Mobility	0.85
School: Using educational Materials	0.88
School: Communication	0.83
HCLA: Household activities	0.84
HCLA: Shopping/managing money	0.82
HCLA: Managing daily schedule	0.84
HCLA: Using transportation	0.79
HCLA: Work activities	0.86
Variance explained	88.7%

*CASP* Child and Adolescent Scale of Participation, *HCLA* Home and Community Living Activities

### Relationship with HrQoL

We found a moderate positive correlation between the CASP and the KINDL^R^ total scales in the total sample (*n* = 314, r = 0.45, *p*-value< 0.001). The correlation was highest in children with mild conditions (*n* = 169, r = 0.50, *p*-value< 0.001), lower in children with severe conditions (*n* = 43, r = 0.35, p-value = 0.022) and weak in children without chronic health conditions (*n* = 102, r = 0.19, p-value = 0.062). There was an excellent correlation between the CASP total scale and the results for the single questionnaire item covering parental perceived overall social participation (*n* = 308, r = 0.86, p-value< 0.001).

### Test-retest reliability

Test-retest reliability was investigated in 22 children. Of these, 19 had a chronic health condition (e.g. developmental and behavioural problems, epilepsy, cerebral palsy, as well as genetic disorders). ICC for the CASP total scale was r = 0.97 (*p*-value< 0.001). For the CASP subscales, the ICC ranged from r = 0.86 (p-value< 0.001; subscale D) to r = 0.96 (p-value< 0.001, subscale B), thereby indicating very good to excellent test-retest reliability. Reference values and association with disease severity

**Fig. 2:**
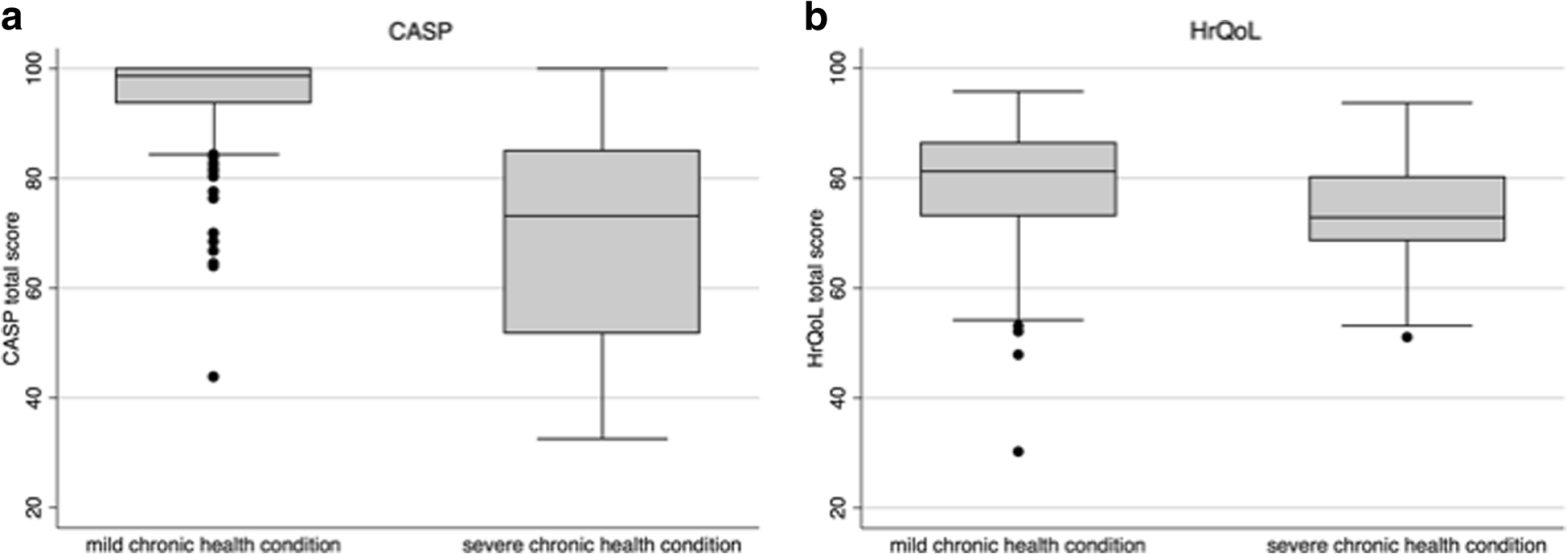
HrQoL and CASP values of chronically ill children in the sample, stratified by disease severity. ^1^CASP = Child and Adolescent Scale of Participation; ^2^HrQoL = Health related Quality of Life

**Fig. 3:**
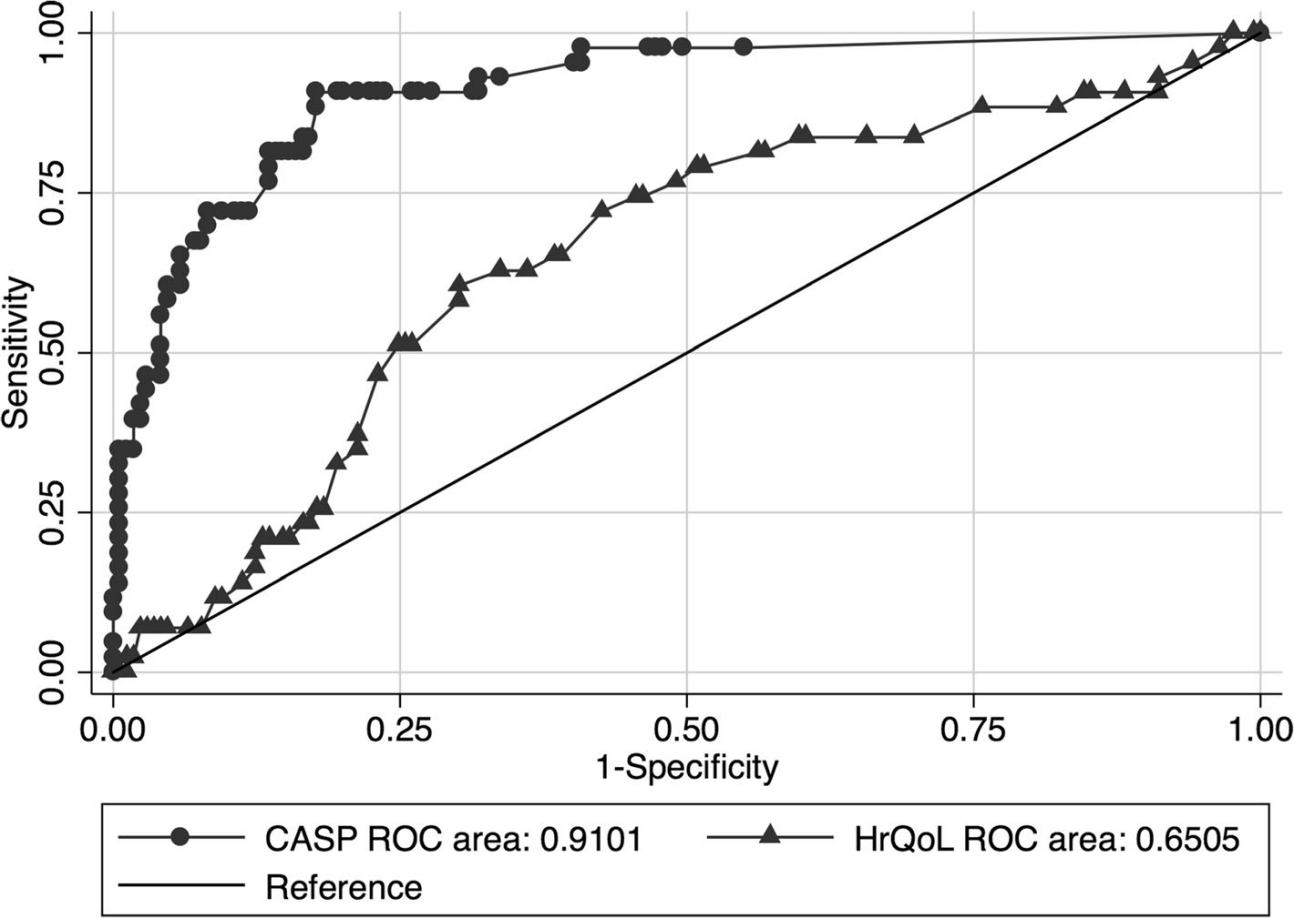
Ability to differentiate between mild and severe chronic disease, for both the CASP and HrQoL. ^1^CASP = Child and Adolescent Scale of Participation; ^2^HrQoL = Health related Quality of Life

Based on the population-based sample of 215 children, mean CASP total score was 98.2 (±5.1), with a high left skewness (− 8.0) and kurtosis (82.6), and the following percentiles (5th: 92.1, 10th: 95, 25th: 98.7, median: 100). Children with mild chronic health conditions had distinctly higher CASP values (mostly> 80) than children with severe chronic health conditions (between 50 and 80: see Fig. 2). In line with Fig. 2, ROC analyses demonstrated that children with mild compared to severe chronic health conditions differed much more in CASP (AUC = .92), than in KINDL (AUC = .75, *p*-value for difference < 0.001, Fig. 3).

Compared to children without chronic health conditions, children with mild conditions had 3.4 points (±1.2; p-value< 0.01) and children with severe conditions had 28.7 points (±1.9; p-value< 0.001) lower values regarding the CASP total scale (Table 4). The differences across groups remained after adjusting for age, sex, and parental education.

**Table 4:** The association between disease severity and the Child and Adolescent Scale of Participation (CASP) based upon multivariate linear regression analysis with sequential adjustments to covariates age, sex, parental education and migrant background

	Model 1 β (SE)	Model 2 β (SE)	Model 3 β (SE)	Model 4 β (SE)
Mild chronic health condition^1^	−4.08^***^ (1.20)	−3.96^**^ (1.19)	−3.61^**^ (1.17)	−3.40^**^ (1.18)
Severe chronic health condition^1^	− 30.16^***^ (1.89)	−29.88^***^ (1.89)	−29.50^***^ (1.85)	− 28.65^***^ (1.92)
Age (years)	0.49 (0.45)	0.49 (0.45)	0.53 (0.44)	0.44 (0.44)
Female^2^		1.86 (1.10)	1.65 (1.08)	1.48 (1.08)
Medium parental education^3^			7.59^†^ (3.09)	6.20 (3.20)
High parental education^3^			10.82^***^ (3.04)	9.27^**^ (3.18)
Migrant background^4^				−2.48 (1.54)
N	277	277	277	277
R^2^	0.50	0.50	0.53	0.54

^1^reference category: without chronic health condition; ^2^reference category: male; ^3^reference category: low educational level; ^4^reference category: no migrant background; † *p* < .05; ** *p* < .01; ***p < .001 *SE* standard error

## Discussion

After translation and back-translation as well as cultural pretesting with consecutive text adaptation, this study investigated psychometric properties of a newly-developed German version of the parent-reported CASP instrument (see Additional file 1) in a large and diverse study sample. We found good to excellent content validity, excellent internal consistency, and good to excellent test-retest reliability of the instrument. We provided reference values obtained in a population-based sample of children and investigated the effects of mild and severe chronic health conditions on CASP results in comparison to a related HrQoL instrument. Finally, a one-item short version that was intentionally added to the CASP to assess overall perceived participation was highly correlated with the CASP total score, thereby indicating the potential value of this specific item on global perceived participation. In summary, we could convincingly demonstrate that the German CASP is sufficiently valid and reliable to be used in clinical health care and future studies.

Our findings on the psychometric properties of the German CASP were similar to those reported for the original CASP by Bedell et al. [11, 15]. Both, internal consistency (α = 0.98 vs. 0.96 [11]) and test-retest reliability (r = 0.97 vs. 0.94 [15]) were found to be exceptionally high. Furthermore, moderate correlation between the CASP total scale and the extent of impairment (r = − 0.58 to − 0.66 [11, 15]) as wells as problems in the physical and social environment (r = − 0.43 to − 0.57 [11, 15]) have been found for the original CASP, which is largely in line with the present results for the correlation between CASP and KINDL^R^ total scale. Our results suggest that there is a positive relationship between social participation and HrQoL, while at the same point both concepts only partly overlap. Thus, participation may not be substituted by HrQoL and both concepts may be simultaneously used as outcomes in studies.

The present exploratory factor analysis showed some differences to the initial validation study. Bedell et al. found a three-factor structure contributing to 63% of the variance explained [11], while our study indicated a single factor-structure contributing to 88.7% of the variance explained, suggesting that all items load on a single latent factor “participation”. We, thus, were not able to replicate the initial three-factor structure. However, from a psychometric viewpoint, a one-factor structure might indicate even higher validity of the CASP with only one underlying concept. As a consequence, we recommend using only the German CASP total scale in clinical care and future studies.

For the first time, we reported reference values for the CASP obtained in a large population-based disability-free sample of regular primary school children. These values may now be used for individual ratings of children. Results showed clear ceiling effects, as > 50% of children showed the highest possible CASP score of 100 (mean: 98). This is in line with a previous report in a sample of 52 children, showing a mean of 97 [11]. As the 10th and 5th percentiles were 95 and 92 in our reference group, we recommend using these cut-offs for judging mildly (< 95) and severely (< 92) impaired social participation if using the German CASP version.

CASP scores differed markedly across disease severity groups. This suggests that a substantial part of the individual burden of disease depends on disease severity. However, other factors affecting the individual burden of disease such as the social context and personality, have not been investigated in our study. However, they might also explain some part of the variance in CASP values.

The CASPs ability to differentiate between mild and severe disease underscores participation as potential outcome in health care research. Our results corroborate previous findings showing relevant differences in participation between children with and without disability [11]. In our study, the differences in CASP scores between severity groups were larger than the differences in HrQoL scores. Hence, participation measures may provide very good differentiation of the impact of the health status on social outcomes in children with different chronic conditions. In comparison to participation, which tries to grasp impairments in social everyday life, HrQoL rather measures the individual’s personal and subjective appraisal of well-being. In children, it generally measures the ability to adapt to challenges, e.g. in the context of chronic health conditions. However, type of condition and disease severity do not show much impact on HrQoL in cross-sectional samples, in particular in HrQoL instruments not covering functional disability [25]. Thus, participation instruments such as the CASP cannot substitute HrQoL instruments, but may rather complement them through the patient-oriented concept and the ability to separate different disease severities.

In summary, the CASP may reveal beneficial effects of medical interventions in a generic way. Future studies should elucidate, whether the CASP is sufficiently sensitive to detect improvements in participation by health interventions. The sensitivity to change might be a challenge especially in children with severe health conditions, as changes attained by therapies might be too small to be detected by a broad and generic measure like the CASP. Another shortcoming of the CASP is that the measurement of participation relies on items that reflect activities in a “normal” everyday life in childhood, without the possibility of individual prioritization or preference-based choice of activities by the child or parent itself. Notwithstanding these concerns, our study suggests that participation as measured by the German CASP may be a helpful instrument for paediatric health services research.

### Strengths and limitations

This study has several strengths and limitations. It is the first study that provides validation data on a comprehensive instrument to measure social participation across a broad age range in German language. For this study, we intentionally sampled participants from three different populations to be able to draw conclusions on the generic value of the CASP. This sampling strategy should increase generalizability to a broad spectrum of settings and diseases in childhood. However, we acknowledge that the convenience sampling impeded a non-responder analysis, thereby limiting overall generalizability.

Another limitation is the age range in our sample. While Bedell [11] assessed validity in a sample of children aged 3–22 years, the age range in this study was only 3–11 years. Therefore, further validation steps of the German CASP in adolescents are definitively needed. On the other hand, social participation in adolescents should be assessed rather by self-reports than by parent-reports. Therefore, it is acceptable that a parent-report instrument is validated only up to an age range of 12 years.

Although we tried to cover a broad range of social and ethnic parental backgrounds in our sample, sufficient German language skills was an important inclusion criterion for study enrolment, which may hamper generalizability of results, in particular, to parents with migrant background and insufficient language skills. In fact, our study was not large enough to demonstrate acceptance and comprehensibility of the instrument (or the concept of social participation at all) in various ethnic groups. This should be acknowledged when the CASP is applied to parents with migrant background.

Similar to Bedell [11], diagnoses within the different disease groups were quite diverse. Thus, particularities and differences between certain conditions were not accounted for. Given that there is no international definition for the identification and severity rating of chronic health conditions, we developed a new algorithm based on the criteria published by Perrin et al. [16] and the ICF principals. However, this attempt had some limitations in acknowledging health care use, but nevertheless seemed to be useful to examine differences in participation across the condition severity spectrum. Further investigations in children with certain diseases or groups of similar diseases might be needed to assess the specific differences in participation. For example, Hwang et al. separated mental from physical disability in their study on the Chinese CASP version [26]).

Both, the participation and the HrQoL results were based on parental reports. For HrQoL, there is evidence for only limited overlap between self-reports and proxy-reports in children [27]. In analogy, self-reports of participation may be more accurate compared to parent-reports in older children and adolescents, which should be addressed in future validation and outcome studies. On the other hand, a positive relationship between participation and HrQoL can be expected to some extent, if the reports are not independent from each other. However, well-validated self-report participation instruments are not yet available for children and we explicitly chose the CASP to be able to cover a broad age range, which is one advantage of this instrument.

At last, we had to cope with a substantial high number of missing data within the CASP due to items that were “not applicable” as predefined for the original CASP. Parents may have misinterpreted the term `age-expected´, especially for the CASP items concerning `home and community living activities´. To simulate everyday health care practice, data were not imputed for most of the analyses, leading to a data loss of up to 33% for regression and correlation analyses. Given that subscales are only valid, if less than 40% of items are missing, we decided to impute missing values for the factor analysis. This could be one reason why we were not able to confirm the 3-factor structure of the original CASP. In the CASP Administration and Scoring Guideline [16], it is recommended “… to not include the non-applicable item in the scoring or to first take the average of the specific subsection and use this as the score for the non-applicable item”. However, studies investigating the effect of various missing imputation techniques on CASP results are missing. In summary, the problem of imputing missing data for the CASP is not satisfactory solved and future validation studies may focus on this specific topic.

## Conclusions

The culturally adapted German version of the original CASP is a valid and reliable instrument to measure social participation in children with and without chronic health conditions. Because of its shortness, its acceptance, and its applicability across a wide range of settings, ages, and disease severities, the German CASP may be used as add-on to HrQoL instruments for outcome assessments in daily paediatric health care and health services research. In addition, reference values are now available, which may provide the fundamental basis for discriminating none from mild and severe impairments in participation. Using instruments for assessing social participation in routine health care will be a critical step towards the implementation of the ICF-CY into daily paediatric practice.

## Additional file

Additional file 1: German Child and Adolescent Scale of Participation (G-CASP) (PDF 870 kb)

## Abbreviations

CAPE/PAC: Children’s Assessment of Participation and Enjoyment (CAPE) and Preferences for Activities of Children (PAC)
CASP: Child and Adolescent Scale of Participation
ICD-10: International Classification of Disease – version 10
ICF-CY: International Classification of Functioning, Disability and Health – Children and Youth
ikidS: Cohort study “**i**ch **k**omme **i**n **d**ie **S**chule”
KINDL: KINDerfragebogen für Lebensqualität (child questionnaire for health-related quality of life)
PEM-CY: Participation and Environment Measure – Children and Youth
