# Supplementary material for: Measuring social participation in children with chronic health conditions: validation and reference values of the child and adolescent scale of participation (CASP) in the German context
Source: BMC Pediatr. 2019 Apr 24;19:125. doi: 10.1186/s12887-019-1495-6 (PMC6482577; doi:10.1186/s12887-019-1495-6)
Supplement: Supplementary file 1 — German Child and Adolescent Scale of Participation (G-CASP) (PDF 870 kb) [file 12887_2019_1495_MOESM1_ESM.pdf]

Elternfragebogen zu Teilhabe und Partizipation (CASP)

Liebe Eltern,

Mit diesem zusätzlichen Fragebogen möchten wir erfassen, wie sich Ihr Kind im Vergleich zu Kindern seines Alters an alltäglichen Aktivitäten beteiligen kann und ob es irgendwelche Einschränkungen gibt. Sie benötigen etwa 10-15 Minuten für die Beantwortung der Fragen. Denken Sie bitte an aktuelle Situationen.

Im Folgenden geht es um die Beteiligung Ihres Kindes an Aktivitäten zu Hause, in der KiTa bzw. Schule, in der näheren Umgebung, Nachbarschaft und in Vereinen. Weiter geht es darum, welche Strategien, Hilfsmittel oder Anpassungen Sie zurzeit eventuell nutzen, damit Ihr Kind besser teilhaben kann.

Es gibt keine richtigen oder falschen Antworten. Kreuzen Sie bitte die Antwort an, die die Beteiligung Ihres Kindes am besten beschreibt. Bei den offenen Fragen am Schluss möchten wir Sie bitten, so gut wie möglich aufzuschreiben, was unternommen wird, damit Ihr Kind besser bei alltäglichen Aktivitäten teilhaben kann.

Bei Ihren Antworten sollten Sie sich immer vorstellen, wie stark die Beteiligung Ihres Kindes ist verglichen mit der Beteiligung anderer Kinder gleichen Alters. Unsere wissenschaftlichen Ergebnisse sollen dazu dienen, Kindern in Zukunft die bestmöglichste Unterstützung im Alltag geben zu können.

 Übersetzt von F. De Bock, Universität Heidelberg, nach dem Original von G. Bedell, 2009, Tufts University USA

Wir bitten Sie, Ihre Einschätzung in folgende Antwortkategorien einzuteilen:

1. **Altersgemäß:** Ihr Kind nimmt an den Aktivitäten im Vergleich zu anderen Kindern gleichen Alters ebenso stark teil (mit oder ohne Hilfestellung bzw. Hilfsmitteln, je nach Alter mit oder ohne Eltern).
2. **Etwas eingeschränkt:** Ihr Kind nimmt an den Aktivitäten im Vergleich zu anderen Kindern seines Alters etwas weniger stark teil (benötigt möglicherweise gelegentliche Betreuung bzw. Hilfe trotz evtl. Hilfsmittel).
3. **Sehr eingeschränkt:** Ihr Kind nimmt an den Aktivitäten im Vergleich zu anderen Kindern seines Alters viel weniger stark teil (benötigt möglicherweise intensive Betreuung bzw. Hilfe).
4. **Nicht in der Lage:** Ihr Kind nimmt im Gegensatz zu anderen Kindern seines Alters an den Aktivitäten fast überhaupt nicht teil.
5. **Nicht zutreffend:** Von Kindern im Alter Ihres Kindes wird das (noch) nicht erwartet.

Gesamtbeurteilung: **Wenn Sie sich alle Bereiche des Alltags Ihres Kindes zusammen anschauen:**
(zu Hause, KiTa/Schule, Vereine, Nachbarschaft etc.)
Wie gut kann Ihr Kind an alltäglichen Aktivitäten teilhaben?

Altersgemäß … ☐ Etwas eingeschränkt … ☐ Sehr eingeschränkt … ☐ Nicht in der Lage … ☐

A. Wie sehr kann sich Ihr Kind im Vergleich zu Kindern gleichen Alters an folgenden Aktivitäten zu Hause beteiligen?

	Altersgemäß	Etwas eingeschränkt	Sehr eingeschränkt	Nicht in der Lage	Nicht zutreffend
1. Geselliges Zusammensein, Spielen oder sonstige Freizeitaktivitäten mit Familienangehörigen zu Hause<br>z. B. • Gesellschaftsspiele, Rollenspiele, Hobbys	☐	☐	☐	☐	☐
2. Geselliges Zusammensein, Spielen oder sonstige Freizeitaktivitäten mit Freunden zu Hause (einschließlich über Telefon).	☐	☐	☐	☐	☐
3. Mitwirkung an familiären Verpflichtungen, Aufgaben und Entscheidungen im Haushalt<br>z. B. • Holen oder Aufräumen bestimmter Gegenstände (bei Aufforderung)<br>• Mithelfen bei kleineren Aufgaben im Haushalt<br>• Einbezug in Entscheidungen über geplante Aktivitäten der Familie	☐	☐	☐	☐	☐
4. Sich selbst versorgen können<br>z. B. • essen, trinken<br>• anziehen<br>• baden, kämmen, auf die Toilette gehen	☐	☐	☐	☐	☐
5. Sich im und um das Haus/Wohnung herum frei bewegen können (einschließlich Garten, Hof)	☐	☐	☐	☐	☐
6. Sich mit anderen Kindern oder Erwachsenen zu Hause unterhalten/ verständigen,(auch (non-) verbal oder mit Hilfsmitteln)	☐	☐	☐	☐	☐

B. Wie sehr kann sich Ihr Kind im Vergleich zu Kindern gleichen Alters an folgenden Aktivitäten <u>im näheren Umfeld bzw. in der Nachbarschaft</u> beteiligen?

	Altersgemäß	Etwas eingeschränkt	Sehr eingeschränkt	Nicht in der Lage	Nicht zutreffend
7. Geselliges Zusammensein, Spielen oder sonstige Freizeitaktivitäten mit Freunden im näheren Umfeld oder in der Nachbarschaft<br>z. B. • Spielplatz, Klettergerüst<br>• Kirmes, Park	☐	☐	☐	☐	☐
8. Organisierte Aktivitäten oder Veranstaltungen im näheren Umfeld oder in der Nachbarschaft<br>z. B. • Kinderturnen, Jugendfeuerwehr, Musikschule<br>• jahreszeitengebundene oder religiöse (Volks-)Feste<br>• Konzerte, Theater, Kino, Restaurantbesuch.	☐	☐	☐	☐	☐
9. Sich in der näheren Umgebung oder in der Nachbarschaft fortbewegen (laufen/gehen)<br>z. B. • in öffentlichen Parks und Gebäuden<br>• Restaurants, Kinos<br>(*Bitte denken Sie vor allem daran, wie Ihr Kind sich eigenständig fortbewegen kann, nicht daran, welche Verkehrsmittel es nutzt.*)	☐	☐	☐	☐	☐
10. Sich mit anderen Kindern oder Erwachsenen im näheren Umfeld oder in der Nachbarschaft unterhalten/verständigen	☐	☐	☐	☐	☐

C. Wie sehr kann sich Ihr Kind im Vergleich zu Kindern gleichen Alters an folgenden Aktivitäten <u>in der Schule bzw. in der KiTa</u> beteiligen?
Sie können sich hier auch auf Informationen der Lehrer beziehen.

	Altersgemäß	Etwas eingeschränkt	Sehr eingeschränkt	Nicht in der Lage	Nicht zutreffend
11. Aktivitäten im Unterricht mit anderen Kindern seiner Klasse/Gruppe in der Schule / KiTa<br>z. B. • sich melden<br>• abwarten<br>• an Regeln halten	☐	☐	☐	☐	☐
12. Geselliges Zusammensein, Spielen oder sonstige Freizeitaktivitäten mit anderen Kindern in der Schule bzw. in der KiTa<br>z. B. • Pausenaktivitäten<br>• Sport, Hobbys<br>• Basteln, Malen<br>• Theaterspielen, AGs	☐	☐	☐	☐	☐
13. Fortbewegung und Mobilität in der Schule bzw. KiTa in Bereichen, die für alle Kinder gleichen Alters zugänglich sind<br>z. B. • Gruppenraum, Turnraum, Außenbereich, Spielplatz<br>• Toilette, Pausenhof, Cafeteria, Bücherei, Kiosk	☐	☐	☐	☐	☐
14. Spiel- und Lernmaterialien nutzen, die anderen Kindern der gleichen Klasse zugänglich sind<br>z. B. • (Bilder-) Bücher, Computer<br>• Stühle, Schreibtische, z.B. wenn diese für Ihr Kind angepasst worden sind	☐	☐	☐	☐	☐
15. Sich mit anderen Kindern oder Erwachsenen in der Schule bzw. KiTa unterhalten/verständigen	☐	☐	☐	☐	☐

**D. Wie sehr kann sich Ihr Kind im Vergleich zu Kindern gleichen Alters an <u>folgenden Aufgaben</u> zu Hause oder im näheren Umfeld beteiligen?**	Altersgemäß	Etwas eingeschränkt	Sehr eingeschränkt	Nicht in der Lage	Nicht zutreffend
16. Aufgaben im Haushalt erledigen<br>z. B. • Aufräumen, Tisch decken, Mahlzeiten zubereiten<br>• Wäsche waschen, Geschirr spülen, Spülmaschine ein- oder ausräumen.	☐	☐	☐	☐	☐
17. Einkaufen und Umgang mit Geld.	☐	☐	☐	☐	☐
18. Tagesablauf selbstständig einhalten und planen<br>z. B. • alltägliche Aufgaben erledigen<br>• selbstständiges Organisieren und Anpassen des Tagesablaufs an neue Gegebenheiten	☐	☐	☐	☐	☐
19. Nutzen öffentlicher Verkehrsmittel oder anderer Fortbewegungsmittel<br>z. B. • zur / von der Schule<br>• für Freizeitaktivitäten	☐	☐	☐	☐	☐
20. Aktivitäten, für die das Kind zu Hause, im Schul- oder KiTa-Alltag zuständig ist<br>z. B. • Fertigstellung von Aufgaben<br>• Pünktlichkeit, Anwesenheit<br>• Kooperation mit Lehrern, Erziehern, Hausaufgaben pünktlich abgeben<br>• aufräumen	☐	☐	☐	☐	☐

Es folgen drei Fragen, bei denen wir Sie darum bitten, im Freitext selbst eine Antwort zu schreiben (es reichen Stichpunkte).

21. Was hilft Ihrem Kind dabei, sich an alltäglichen Aktivitäten zu beteiligen? (z. B. wie sich Ihr Kind selbst hilft; was andere Personen wie Familienangehörige, Freunde, Erzieher oder medizinische Fachkräfte tun, um Ihrem Kind zu helfen)

____

____

22. Benutzt Ihr Kind zurzeit irgendwelche Hilfen, die seine Beteiligung erleichtern?
(z. B. einen Duschsitz, Rollstuhl, Brille, Hörgeräte, orthopädische Einlagen, einen Tagesplaner, einen Computer)

____

____

23. Wurde bei Ihnen zu Hause, im näheren Umfeld oder in der Schule/KiTa irgendetwas verändert, wodurch Ihr Kind besser teilhaben kann?
(z. B. Möbel oder Materialien umgeräumt, die Beleuchtung oder der Geräuschpegel angepasst, eine Rampe angebracht)

____

____

Herlichen Dank für Ihre Unterstützung!
